# Pharmacokinetic/Pharmacodynamic Dosage Individualization of Suppressive Beta-Lactam Therapy Administered by Subcutaneous Route in Patients With Prosthetic Joint Infection

**DOI:** 10.3389/fmed.2021.583086

**Published:** 2021-03-31

**Authors:** Sylvain Goutelle, Anne Conrad, Cécile Pouderoux, Evelyne Braun, Frédéric Laurent, Marie-Claude Gagnieu, Sabine Cohen, Jérôme Guitton, Florent Valour, Tristan Ferry

**Affiliations:** ^1^Hospices Civils de Lyon, Groupement Hospitalier Nord, Service de Pharmacie, Lyon, France; ^2^Univ Lyon, Université Lyon 1, ISPB, Faculté de Pharmacie de Lyon, Lyon, France; ^3^Univ Lyon, Université Lyon 1, UMR CNRS 5558, Laboratoire de Biométrie et Biologie Evolutive, Villeurbanne, France; ^4^Centre interrégional de référence pour la prise en charge des infections ostéo-articulaires complexes (CRIOAc Lyon), Hospices Civils de Lyon, Lyon, France; ^5^Service des maladies infectieuses et tropicales, Hôpital de la Croix-Rousse, Hospices Civils de Lyon, Lyon, France; ^6^Université Claude Bernard Lyon 1, Lyon, France; ^7^CIRI—Centre International de Recherche en Infectiologie, Inserm U1111, Université Claude Bernard Lyon 1, CNRS, UMR5308, Ecole Normale Supérieure de Lyon, Univ Lyon, Lyon, France; ^8^Institut des Agents Infectieux, Laboratoire de bactériologie, Centre National de référence des staphylocoques, Hôpital de la Croix-Rousse, Hospices Civils de Lyon, Lyon, France; ^9^Hospices Civils de Lyon, Groupement Hospitalier Sud, Service de Biochimie et Biologie Moléculaire, UM Pharmacologie—Toxicologie, Lyon, France

**Keywords:** prosthetic-joint infection, antimicrobial therapy, pharmacodynamics, pharmacokinetics, beta-lactam, subcutaneous administration

## Abstract

Suppressive parenteral antibiotic therapy with beta-lactams may be necessary in patients with Gram-negative bone and joint infection (BJI). Subcutaneous drug administration can facilitate this therapy in outpatient setting, but there is limited information about this practice. We have developed an original approach for drug dosing in this context, based on therapeutic drug monitoring (TDM) and pharmacokinetic/pharmacodynamic (PK/PD) principles. The objective of this study was to describe our approach and its first results in a case series. We analyzed data from patients who received suppressive antibiotic therapy by subcutaneous (SC) route with beta-lactams as salvage therapy for prosthetic joint infection (PJI) and had TDM with PK/PD-based dose adjustment. Ten patients (six women and four men with a mean age of 77 years) were included from January 2017 to May 2020. The drugs administered by SC route were ceftazidime (*n* = 4), ertapenem (*n* = 4), and ceftriaxone (*n* = 2). In each patient, PK/PD-guided dosage individualization was performed based on TDM and minimum inhibitory concentration (MIC) measurements. The dose interval could be prolonged from twice daily to thrice weekly in some patients, while preserving the achievement of PK/PD targets. The infection was totally controlled by the strategy in nine out the 10 patients during a median follow-up of 1,035 days (~3 years). No patient acquired carbapenem-resistant Gram-negative bacteria during the follow-up. One patient presented treatment failure with acquired drug resistance under therapy, which could be explained by late MIC determination and insufficient exposure, retrospectively. To conclude, our innovative approach, based on model-based TDM, MIC determination, and individualized PK/PD goals, facilitates, and optimizes suppressive outpatient beta-lactam therapy administered by SC route for PJI. These encouraging results advocate for larger clinical evaluation.

## Introduction

Prolonged suppressive antimicrobial therapy (SAT) is necessary in some patients with prosthetic joint infection (PJI). This may be the only option to control the infection in patients for whom surgical removal of the prosthesis cannot be performed for various reasons (1). Those patients are often old, with multiple co-morbidities. In most cases, SAT is administered in outpatient setting. Oral antibiotics active against Gram-positive bacteria are the most frequently prescribed drugs in this indication (1). However, in case of infections caused by fluoroquinolone- and cotrimoxazole-resistant Gram-negative pathogens, parenteral administration may be necessary with beta-lactams usually used intravenously such as ceftriaxone, ceftazidime, and even ertapenem.

In case of prolonged parenteral antibiotic therapy with injectable beta-lactams, important questions are the dosage regimen that should be administered and the route of administration. Conventional dosing of beta-lactams consists on daily (e.g., ertapenem and ceftriaxone) or multiple daily intravenous administrations (e.g., ceftazidime). The dosage regimen is governed by the pharmacokinetic/pharmacodynamic (PK/PD) properties of those agents that often have a short half-life and exhibit time-dependent antibacterial effect (2). Thus, frequent administration of beta-lactam is thought to be necessary to maintain antibiotic concentration above the minimum inhibitory concentration (MIC) over a sufficient time between two administrations.

Frequent administration of intravenous (IV) drugs has several limitations in the outpatient setting. Long-term venous access should be maintained and requires specific care. Frequent IV administration is laborious for nurses, uncomfortable for patients, and costly. Spacing drug administration is desirable in this setting, but it should respect the PK/PD requirements of each drug to ensure treatment efficacy. It has been shown that the subcutaneous (SC) route may facilitate drug administration in patients compared with IV route, while preserving the PK/PD objectives of beta-lactams (3–8). Combining infrequent administration and SC route could be a way to facilitate prolonged suppressive outpatient therapy with beta-lactams, but there is limited information on this practice.

The objective of this work was to report the principles and first results of our salvage dosing approach for suppressive outpatient SC antibiotic therapy with beta-lactams based on PK/PD monitoring.

## Methods

### Data Collection and Patients' Therapy

We analyzed data from patients who received suppressive antibiotic therapy by SC route with beta-lactam as salvage therapy and had therapeutic drug monitoring with PK/PD-based dose adjustment from January 2017 to May 2020 in our reference center for bone and joint infection called CRIOAc Lyon (http://www.crioac-lyon.fr). Part of the data have been reported in a previous article that focused on safety and outcome (7). The present study focuses on PK/PD and dosage individualization, in patient with PJI. All patients gave their consent to be included in the Lyon BJI cohort study that is registered on the website clinicaltrial.gov (NCT02817711). Collecting data on the efficacy and safety of off-label antibiotic in BJI is one of the objectives of this cohort study.

Three beta-lactams were used as suppressive therapy by SC route: ertapenem, ceftriaxone, and ceftazidime. Subcutaneous administration of those three drugs is still off-label in France but is supported by several clinical reports and studies (6, 7, 9–11). The decision of suppressive antibiotic therapy was taken by a multidisciplinary team including infectious disease physicians, surgeons, and microbiologists. Parenteral drugs were used when no oral drug could be administered because of the pathogen's resistance profile and/or polymicrobial infection and/or history of drug-related adverse events. The SC route was selected in order to facilitate outpatient care and the patient's acceptance of prolonged therapy, especially as suppressive intravenous therapy was not considered as feasible (benefit/risk ratio was considered in favor of the SC instead of intravenous administration). SC administration consisted in a 30–45-min gravity infusion of the diluted antibiotic (50 ml of isotonic saline) via a disposable butterfly needle inserted in the anterior side of the thigh or in the abdominal flank. Patients were followed-up at least every month at CRIOAc Lyon. The suppressive parenteral antibiotic therapy was started during hospitalization, after conventional primary intravenous antimicrobial therapy, based on microbiology data (type of bacteria and drug susceptibility). The initial dosing regimen was conventional with daily or multiple daily administrations depending on the beta-lactam considered and patients' characteristics. As for all patients receiving a prolonged beta-lactam in our institution, screening for rectal carriage for third cephalosporin-resistant of carbapenem-resistant *Enterobacteriaceae* was performed during the follow-up.

### Pharmacokinetic/Pharmacodynamic Dosage Individualization

Therapeutic drug monitoring (TDM) of the drug was first performed under conventional dosing, at the steady state. Blood samples were obtained during a planned follow-up visit in the BJI center. A typical PK profile included three samples: one pre-dose (trough or *C*_min_), one 30 min after the end of the SC infusion (*C*_max_), and one about 5–6 h after the end of the administration. The sampling times were precisely recorded for each patient, as well as body weight and renal function at the date of TDM. Drug concentrations of ertapenem, ceftriaxone, and ceftazidime were measured by validated liquid chromatography methods that are available in routine analysis in our institution.

The results were then analyzed by PK modeling. We used the BestDose^TM^ software to perform Bayesian estimation of individual PK parameters (e.g., clearance and volume of distribution) in each patient (12). Once the model had been fit to data and provided acceptable results, it was used to simulate a future dosing regimen. Future dosing regimens with standard and increased dosing interval (e.g., every 48 h or three administrations per week) were examined. The achievement of the PK/PD objective was calculated based on predicted concentrations and the MIC of the pathogen identified in bone samples, when available. When the MIC of the bacteria was not available, we used the MIC distribution of the bacteria provided by EUCAST. For beta-lactam, the usual objective is to maintain free (i.e., unbound to plasma protein) concentrations above the MIC (*f* T > MIC) over 50% to 100% of the dosing interval (2). In patients treated for BJI, this objective may be revised according to bone penetration. For ceftriaxone, available data suggest that bone to plasma concentration ratios are similar to the plasma free fraction of the drug, about 5–10% (13). For ertapenem, Boselli et al. (14) reported bone to plasma concentration ratios ranging from 0.1 to 0.4, which is higher than the free fraction in plasma (5–10%). For ceftazidime, Leigh et al. (15) reported mean bone-to-serum concentration ratios ranging from 0.20 to 0.30, depending on the site and bone tissue. This is lower than ceftazidime free fraction in plasma that is about 80%. We considered the worst-case scenario in terms of bone penetration for each agent and set the target plasma concentration to be achieved as 10 to 20 × MIC for ertapenem and ceftriaxone (i.e., assuming that bone concentration is equal to the free fraction in plasma) and 5 × MIC for ceftazidime. An individualized drug dosage, with increased dosing interval, was suggested to the clinicians whenever possible. The achievement of the PK/PD objectives was controlled by TDM and modeling on subsequent visits when feasible.

## Results

Ten patients with PJI received SC suppressive antibiotic therapy and had dosage based on TDM and PK/PD on the study period. This case series included six women and four men, with median (min–max) age, body weight, and creatinine clearance of 77 years (63–83), 77 kg (68–115), and 62 ml/min (35–118), respectively. Their characteristics are shown in Table 1, as well as the PK/PD results and dosage adjustment. The drugs administered as suppressive therapy were ceftazidime (*n* = 4), ertapenem (*n* = 4), and ceftriaxone (*n* = 2). In each patient, PK/PD-guided dosage individualization was performed, with changes in drug amount and/or dose interval based on TDM and MIC measurements.

**Table 1:** Characteristics of patients who received SC outpatient beta-lactam therapy and model-based TDM results.

**Patient number (sex)**	**Age (years)^a^**	**Type of PJI**	**Weight (kg)^a^**	**CL_**CR**_ (ml/min)^a^**	**Drug monitored**	**Targeted pathogen**	**Pathogen MIC (target concentration) in mg/l**	**TDM #1**		**TDM #2**		**TDM #3**	
								**Dosage (route)**	***C*_**min**_ in mg/l (time >target)**	**Dosage (route)**	***C*_**min**_ in mg/l (time >target)**	**Dosage (route)**	***C*_**min**_ in mg/l (time >target)**
1 (M)	83	Hip	74	60	Ceftazidime	*E. coli*	0.5 (2.5)	8 g/24 h (IV, CI)	NA	2 g/24 h (SC)	5.6 (100%)	1 g/24 h (SC)	2.8 (100%)
2 (M)	81	Knee	110	58	Ceftazidime	*P. aeruginosaE. coli*	0.75 (3.75)^b^	2 g/24 h (SC)	4.9 (100%)	2 g TW (SC)	<1 (50%)	–	–
3 (M)	69	Knee	80	96	Ceftazidime	*P. aeruginosa*	2 (10)	3 g/12 h (SC)	22.4 (100%)	3 g/24 h (SC)	2.0 (58%)	–	–
4 (M)	78	Hip	73	35	Ceftazidime	*P. aeruginosa*	2 (10)	1 g/12 h (SC)	20.7 (100%)	1 g/24 h (SC)	7.0 (83%)	1 g Mon	2.7 (40%)
												1 g Wed	
												2 g Fri (SC)	
5 (F)	75	Knee	74	118	Ertapenem	*E. cloacae*	0.38 (7.6)^c^	1 g/24 h (SC)	3.7 (NA)	1 g Mon	<1 (37%)	–	–
										1 g Wed			
										2 g Fri (SC)			
6 (F)	78	Hip	68	90	Ertapenem	*E. cloacae*	0.064 (1.28)	1 g/24 h (SC)	3.9 (100%)	1 g/48 h (SC)	2.7 (100%)	–	–
7 (F)	75	Hip	90	61	Ertapenem	*E. coli*	≤0.5 (10)^d^	1 g/12 h (SC)	43.9 (100%)	1 g/24 h (SC)	17.0 (100%)	1 g Mon	3.5 (55%)
												1 g Wed	
												2 g Fri (SC)	
8 (F)	63	Hip	80	63	Ertapenem	*E. asburiae*	0.032 (0.64)	1 g/12 h (SC)	35.4 (100%)	1 g/24 h (SC)	14.2 (100%)	1 g TW (SC)	2.3 (100%)
9 (F)	80	Knee	70	56	Ceftriaxone	*S. marcescens*	≤1 (16)^e^	2 g/24 h (SC)	71.7 (100%)	1 g Mon	20.3 (100%)	–	–
										1 g Wed			
										2 g Fri (SC)			
10 (F)	74	Knee	115	80	Ceftriaxone	*E. coli*	0.023 (0.5)	2 g/12 h (SC)	61.7 (100%)	2 g TW	6.6 (100%)	–	–

a *Values at the time of the first TDM*.

b *Both bacteria had the same MIC*.

c *The MIC was not available when thrice weekly dosing was started after the first TDM results. Later, it was reported as 0.38 mg/l (see main text)*.

d *The MIC was initially not available for this patient. We assumed a maximal MIC of 0.5 mg/l, based on the MIC distribution from EUCAST. Thereafter, the initial MIC was measured at 0.032 mg/l. The MIC measured on samples collected after relapse was 0.023 mg/l*.

e *The MIC was not measured for this patient and there is no epidemiological cut-off (ECOFF) defined for Serratia marcescens with ceftriaxone. We considered the ECOFF of cefotaxime (1 mg/l) provided by EUCAST*.

*BJI, bone and joint infection; CI, continuous infusion; Fri, Friday; MIC, minimum inhibitory concentration; Mon, Monday; NA, not applicable; PJI, prosthetic joint infection; TDM; therapeutic drug monitoring, TW, thrice weekly; Wed, Wednesday*.

**Figure 1:**
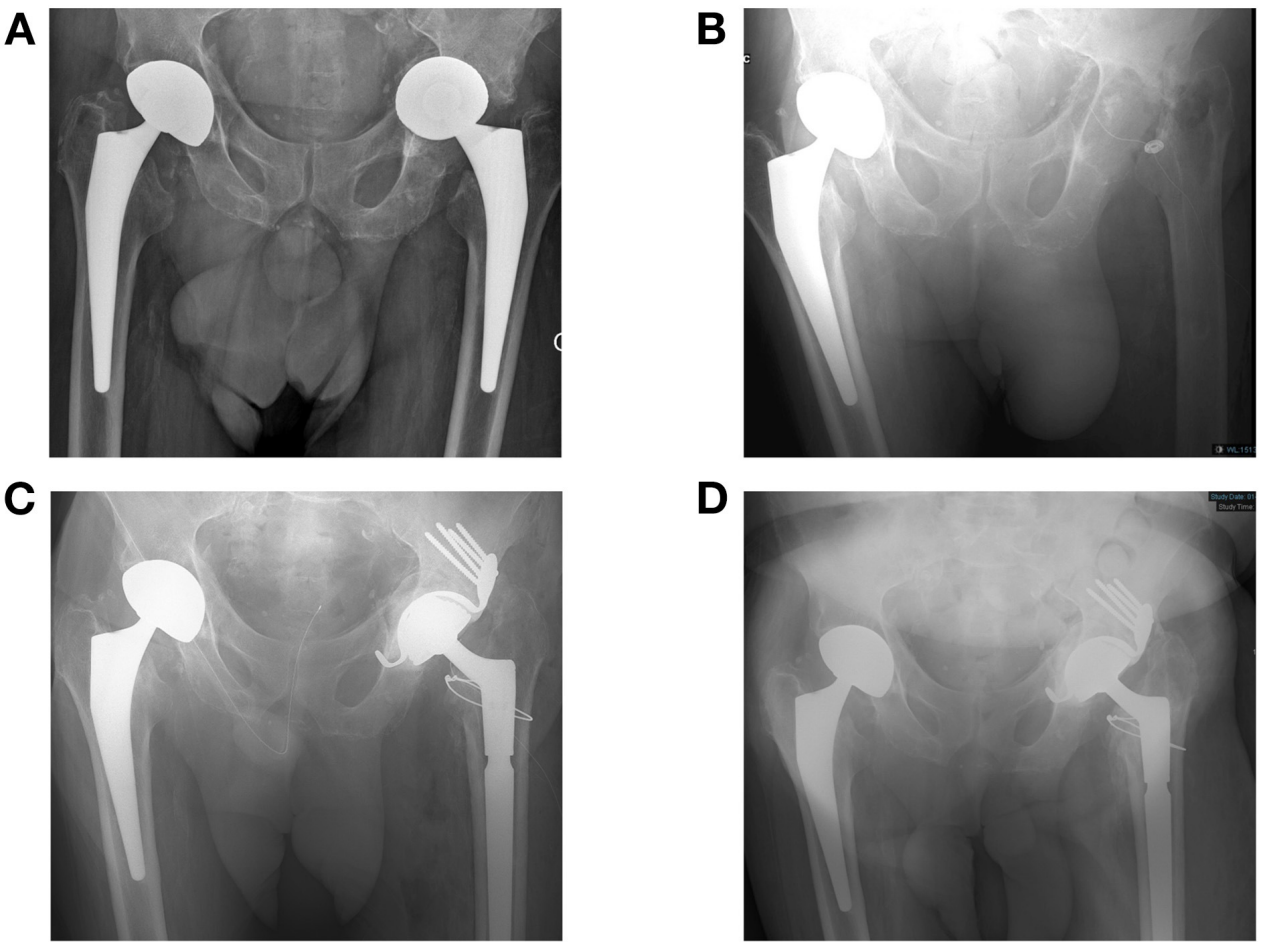
Clinical description of the patient #4, a 77-year-old man. He had a past history of anal cancer and congestive heart failure with arrhythmia. Right and left hip prostheses were implanted in 2013 and 2014, respectively, both following femoral head fracture. As prosthesis loosening occurred with migration of the prosthesis in the pelvis **(A)**, a prosthetic joint infection (PJI) of the left hip was suspected. Explantation was performed in 2017 **(B)**, revealing *P. aeruginosa* chronic infection. Unfortunately, the strain was resistant to ciprofloxacin, but remained susceptible to ceftazidime. Intravenous (IV) ceftazidime was administered after explantation and stopped 15 days before reimplantation. At the time of reimplantation 3 months later, a complex acetabular reconstruction with the Burch–Schneider antiprotrusio cage and allografts was performed **(C)**, without any occurrence of loosening during the prolonged follow-up of 2 years **(D)**. As the cultures were still positive with persistence of *P. aeruginosa* in culture with the same susceptibility, IV ceftazidime 2 g/8 h was prescribed again. The dose was then reduced to 1 g/12 h as chronic kidney injury occurred (creatinine clearance 30 ml/min), before performing the first therapeutic drug monitoring (TDM) 6 months after the reimplantation, after switch from IV to SC ceftazidime 1 g/12 h. The outcome was favorable with a total control of the infectious disease (i.e., without occurrence of any sign of infection) during the follow-up. Unfortunately, the patient died ~2 years after de reimplantation (727 days) following trauma and hemorrhagic shock.

**Figure 2:**
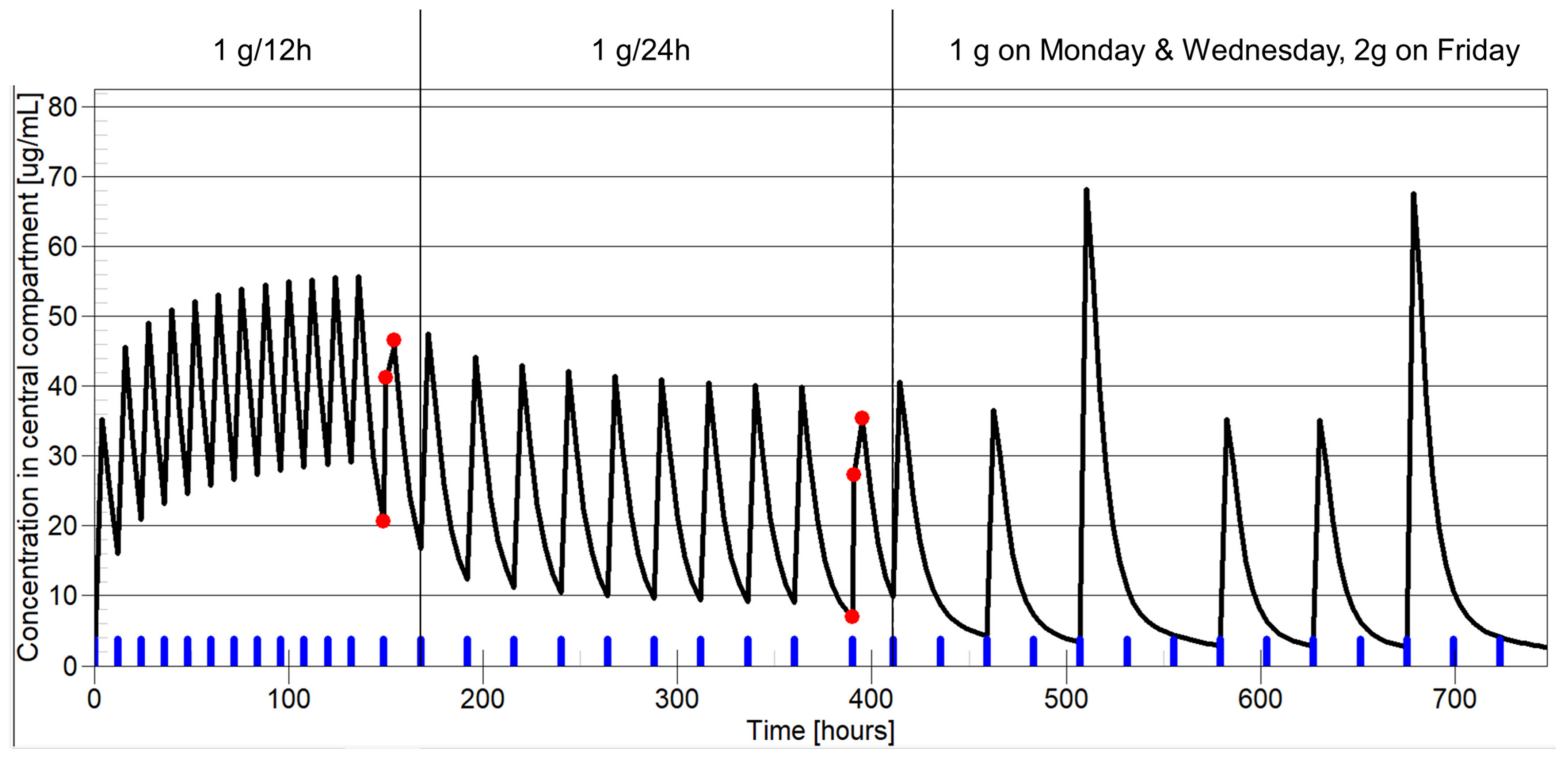
Example of dosage individualization based on pharmacokinetic/pharmacodynamic (PK/PD) in patient #4, treated with suppressive ceftazidime for persistent *P. aeruginosa* PJI. The *x*-axis shows the time, the *y*-axis represents ceftazidime plasma concentration. Of note, this is not the real time of drug therapy. The time scale has been altered to show the three dosage periods on the same plot. The blue marks on the *x*-axis show drug administrations. The red dots represent the patient-measured ceftazidime concentrations. The black line represents model prediction. The vertical line separates the three dosage periods: 1 g/12 h, 1 g/24 h, and 1 g on Monday and Wednesday + 2 g on Friday. On the first TDM occasion, under a dosage of 1 g/12 h, the measured ceftazidime *C*_min_ was 20.7 mg/l, well above the target concentration of 10 mg/l (5 × MIC) for this patient. The model predicted that a dosage of 1 g/24 h would result in *C*_min_ of 8.2 mg/l and 88% of time above the target level. The dosage was adjusted as suggested. TDM was performed a second time, 2 months later, under a dosage of 1 g/24 h. The measured ceftazidime *C*_min_ was 7 mg/l, in good agreement with the model prediction 2 months before. The model predicted that a dosage of 1 g on Monday and Wednesday and 2 g on Friday would result in *C*_min_ of 2.7 mg/l and 40% of time spent above the target level of 10 mg/l (5 × MIC).

An illustrative case of our dosing approach is that of a 77-year-old man who had a chronic PJI of the hip (patient #4; Figure 1). The pathogen identified was *Pseudomonas aeruginosa* with a measured MIC of 2 mg/l for ceftazidime. At the time of the first TDM, he had renal impairment with estimated glomerular filtration rate of 34 ml/min/1.73 m^2^. His weight was 73 kg. He was initially administered SC ceftazidime 1 g/12 h. The target concentration for this patient was set at 10 mg/L (5 × MIC). Lowering the number of administration was examined to facilitate outpatient therapy. Figure 2 summarizes how his dosage regimen was changed from 1 g/12 h to a thrice weekly regimen based on TDM and PK/PD modeling.

**Figure 3:**
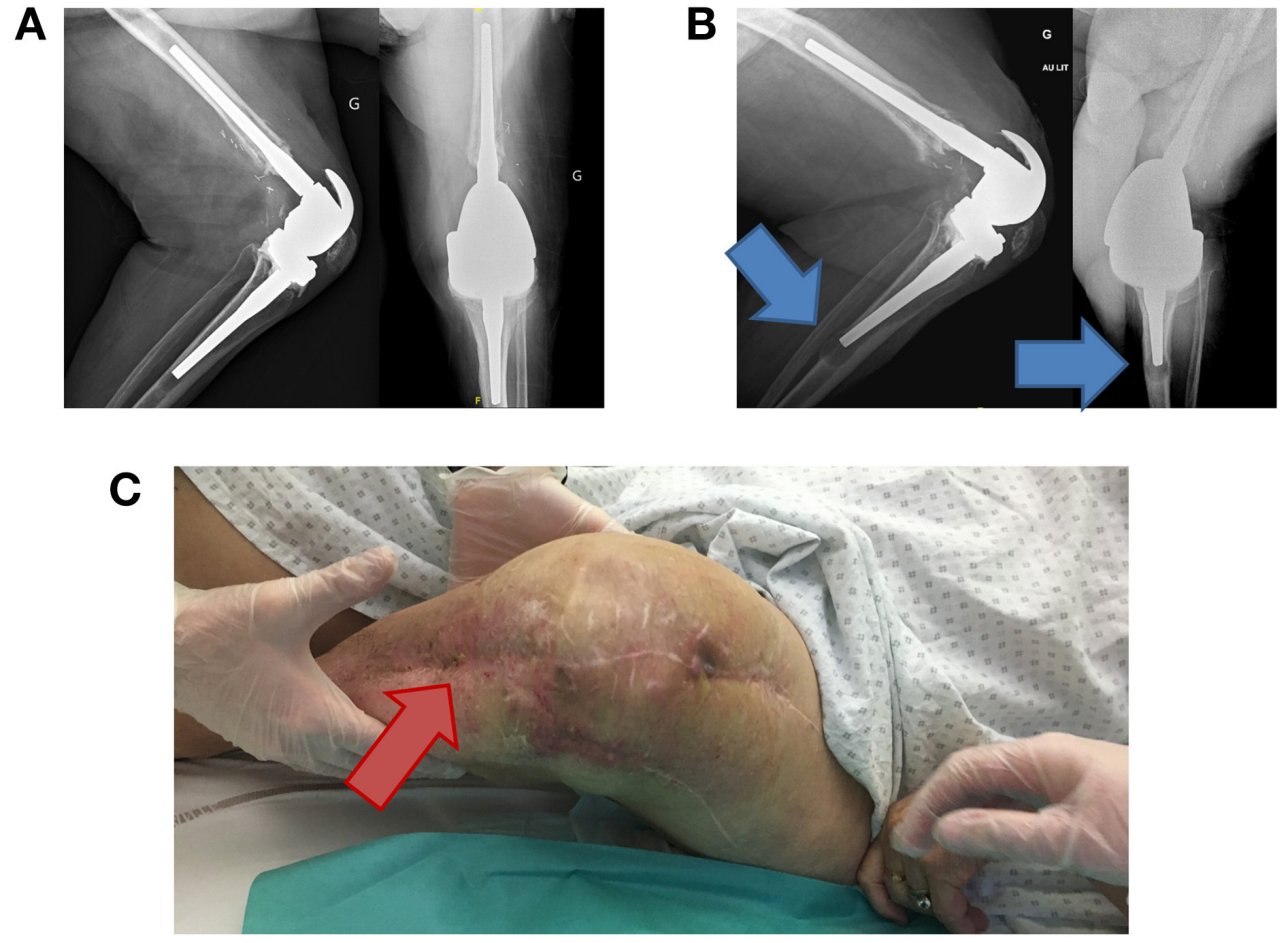
Clinical description of patient #5, a 75-year-old woman. She had a past history of diabetes, Parkinson's disease, and hypertension. A left knee prosthesis was implanted in 2009. In 2017, she experienced a distal femoral fracture requiring osteosynthesis and then debridement for acute infection. As a pseudarthrosis occurred, a two-stage exchange was performed. *Enterobacter cloacae* producing extended-spectrum beta-lactamases (ESBL) was found, and imipenem was prescribed and was stopped after the reimplantation. Unfortunately, the patient developed signs of acute infection and a new debridement revealed *Pseudomonas aeruginosa* and *Candida albicans* superinfection, with persistence of the ESBL *Enterobacter cloacae*. The initial therapy included IV imipenem, oral ciprofloxacin, and oral fluconazole. After 6 weeks, imipenem was replaced by IV ertapenem (1 g/12 h), and irreductible flessum persisted with mild prosthesis loosening on X-ray **(A)**. Ciprofloxacin and fluconazole were stopped after 12 weeks and 6 months, respectively. Six months after surgery, ertapenem administered as 1 g/24 h by SC route was continued as suppressive therapy targeting the multidrug-resistant *Enterobacter cloacae*. Unfortunately, prosthesis loosening **(B)** and purulent discharge occurred **(C)** (the red arrow points to the fistula from which purulent discharge occurred) revealing the persistence of the ESBL *Enterobacter cloacae* into the joint, despite ertapenem therapy. It became resistant to ertapenem.

**Figure 4:**
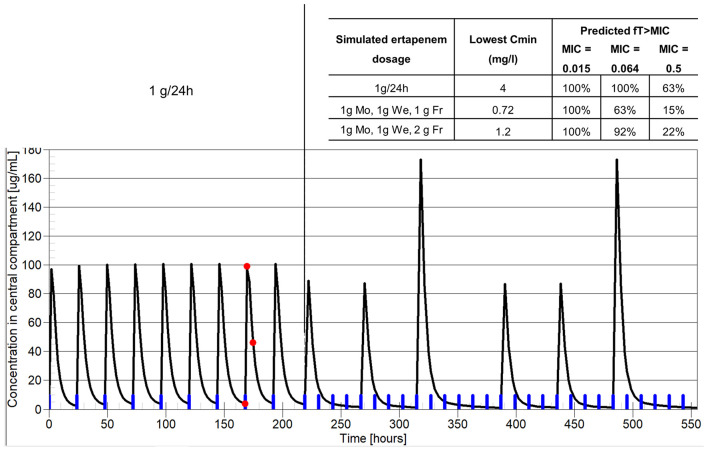
Example of dosage individualization based on PK/PD in patient #5, treated with suppressive ertapenem for a persistent *E. cloacae* PJI. The *x*-axis shows the time and the *y*-axis represents ertapenem plasma concentration. Of note, this is not the real time of drug therapy, as past therapy before TDM was much longer. The blue marks on the *x*-axis show drug administrations. The red dots represent the patient-measured ertapenem concentrations. The black line represents model prediction. The vertical line separates the past therapy with 1 g/24 h and predicted future therapy. The inserted table shows the predicted *C*_min_ and PK/PD objective for three candidate dosage regimens and three possible MIC values.

A second case illustrates the importance of MIC in the dosing decision (patient #5, Figure 3). This was a 75-year-old woman who had a complicated chronic PJI of the knee. Six months after surgery, ertapenem administered as 1 g/24 h by SC route was continued as suppressive therapy targeting the multidrug resistant *Enterobacter cloacae*. The patient had no signs of uncontrolled infection at this time but experienced a poor functional outcome with irreductible flessum and mild lucencies on X-ray (Figure 3A). This targeted bacteria was reported to be susceptible to ertapenem, but the MIC was not available and it was unknown when TDM was performed. TDM was first performed about 4 months after SC ertapenem was started, as the patient was inquiring about the possibility of less frequent SC injections. At the time of TDM, the patient weighted 74 kg and had creatinine clearance of 118 ml/min. Figure 4 shows the estimated PK profile obtained after Bayesian estimation of PK parameters based on three measured concentrations, the alternative dosage adjustment examined, and the predicted value of the PK/PD objective (*f* T > MIC). The target concentration was set at 20 times the MIC, as explained above. As the MIC was unknown, we considered three putative MIC values based on the ertapenem MIC distribution of *Enterobacter cloacae* provided by EUCAST: a low MIC of 0.015 mg/l, an intermediate MIC of 0.064 mg/l, and a high MIC of 0.5 mg/l. The achievement of the PK/PD target under thrice weekly dosage regimens strongly depended on the MIC. The results were acceptable for MIC ≤ 0.064, with *f* T > MIC greater than 40% and up to 100%. However, the exposure was clearly not sufficient for the high MIC. Of note, 1 g/24 h was associated with more favorable PK/PD, with *f* T > MIC of about 60% for a MIC of 0.5 mg/l. Based on this simulation, the dosage of SC ertapenem was adjusted with 1 g on Monday and Wednesday and 2 g on Friday. Unfortunately, 7 months after this dosage adjustment, the patient showed treatment failure, with total prosthesis loosening (Figure 3B) and purulent discharge with acquired resistance of *Enterobacter cloacae* to ertapenem. The MIC of the original strain that was finally retrieved was at 0.38 mg/l, a high value associated with insufficient *f* T > MIC of the thrice weekly regimen, retrospectively.

Except for this latter patient, in whom the failure was predictable *a posteriori*, the infection was totally controlled by the strategy in nine out the 10 patients during a median follow-up of 1,035 days (~3 years) (extreme values 251 and 1,664 days; interquartile range 372–1,291 days); eight of them were followed >2 years without any recurrence, except for one patient (patient #7) in whom ertapenem was stopped when COVID-19 was diagnosed. Unfortunately, 2 weeks after the withdrawal of ertapenem, the patient presented a clinical failure with the same pathogen (*E. coli*) that remained susceptible to ertapenem (MIC = 0.023 mg/l), demonstrating that our model-based TDM SC outpatient beta-lactam therapy was efficient as long as the treatment was continued. Concerning the potential acquisition of resistant bacterial carriage in the gut microbiota, nine patients were already colonized with 3^rd^ generation cephalosporin-resistant Gram-negative bacteria before suppressive therapy, and one of them lost it during the follow-up. One patient never acquired any resistant bacterial carriage. No patient acquired carbapenem-resistant Gram-negative bacteria during the follow-up.

## Discussion

Prolonged suppressive outpatient parenteral antimicrobial therapy is demanding for patients and health care professionals. This case series illustrates how the route of administration and the dosage regimen can be individualized to facilitate this therapy. Our approach for route and dosage individualization of beta-lactam in patients with PJI is basically based on four principles: SC administration; drug TDM; pathogen MIC determination; and model-based, goal-oriented dose adjustment.

First, the subcutaneous route facilitates drug administration in such setting compared with IV route. The venous access required for IV administration may be difficult to maintain in the long term and is associated with a higher risk of infection (17). The SC route also appears to be preferred by patients, as it reduces discomfort and facilitates home care compared with IV route (18).

From a PK/PD perspective, SC administration is especially interesting for the administration of beta-lactam drugs, as it results in time above the MIC similar if not greater than IV administration of the same dose (3–5, 8). This suggests that SC administration of beta-lactams should be as efficient as IV administration, although there is a dearth of high-level clinical evidence.

The second principle is to perform TDM, with plasma concentration measurement of beta-lactams. In our approach, TDM is key to perform model-based dose adjustment. It is also useful to check that the target concentration is achieved after increasing the dose interval. Of note, TDM of beta-lactam is often performed as a trough-only approach in other settings (16, 19). A trough level is sufficient provided that its value is greater than the target concentration, which is most often a multiple of the MIC, so that the time spent above the target level is 100%. However, in the case of trough concentration lower than the MIC, the result cannot be interpreted. The time spent above the MIC is lower than 100% but remains unknown. Because our goal is to space drug administration for facilitating prolonged antibiotic therapy, we use a different TDM approach with three drug samples. This allows to better estimate the individual PK parameters of the drug with the model (including half-life) and to calculate the time spent above the MIC in all cases, even when it is lower than 100%.

Third, as illustrated in Figure 2, the determination of the pathogen MIC is important in our approach for dosage individualization. Basically, the MIC determines the individual requirements in terms of drug exposure and so determines the therapeutic margin. As shown in Table 1, infrequent administration of beta-lactams, even those with short half-life such as ceftazidime, is possible when the MIC is low, because the PK/PD target (*f* T > MIC of 50–100%) can still be achieved. For example, in patients #6, 8, and 10, the observed *f* T > MIC was still 100% even under thrice weekly dosage. By contrast, when the bacterial MIC is high, increasing the dosage interval is not possible, because *f* T > MIC will be insufficient to ensure efficacy.

It has been suggested elsewhere that the MIC epidemiological cut-off (ECOFF) of the pathogen should be used to interpret TDM results and perform dose adjustment of antibacterials, because the precision of MIC assay is often low (20). We believe that this is not justified in all situations, especially when the measured MIC is much lower than the ECOFF (21). Using the ECOFF for PK/PD-based dose adjustment consists in considering the worst-case scenario and the need for high dosage in all patients infected by a given pathogen. Basically, this assumption preclude PK/PD dosage individualization. By contrast, as shown in Table 1, using the measured MIC permits to set individualized goals in each patient and adjust the dosage to patients' condition and needs.

Our approach for dosage individualization is based on Bayesian PK modeling and dose adjustment. The use of PK models permits to interpret TDM results most efficiently, as one can calculate the individual PK parameters, estimate the value of the PK/PD objective (e.g., *f* T > MIC for beta-lactams), and simulate future dosage regimens achieving the individual target. Bayesian dosing programs outperform empirical and other dose adjustment methods (22, 23). This approach is especially useful to predict the adequacy of infrequent drug administration of beta-lactams in our setting, which would be virtually impossible without models. In our case series, most model predictions have been confirmed by subsequent concentration measurements when the patients were stable (data not shown).

Finally, the tolerance and safety of prolonged suppressive subcutaneous antibiotic therapy is also a major challenge, considering the off-label characteristic of this procedure and the potential risk of acquisition of carbapenem-resistant bacterial carriage in the gut microbiota. All patients received therapy over several months or years, corresponding to ~4,000 SC injections, without any serious adverse event at the site of injection. None of our patients acquired a carbapenem-resistant bacteria detectable in stools during the follow-up, which is reassuring in a safety point of view.

There is a number of limitations in this study. The clinical results should be interpreted cautiously because of the limited sample size. We used conventional PK/PD targets for beta-lactam therapy (*f* T > MIC of 50% to 100%) but those have not been evaluated in patients with PJI. Limited data was available from each patient, as TDM was performed infrequently. The long-term efficacy and safety of subcutaneous suppressive beta-lactam therapy administered by SC route remains to be evaluated in prospective clinical trials.

To conclude, this case series shows that suppressive outpatient beta-lactam therapy administered by SC route in patients with PJI is feasible. We have developed an innovative approach to facilitate and optimize this therapy based on model-based TDM, MIC determination, and individualized PK/PD goals. This approach has shown encouraging results so far for these patients requiring salvage therapy but needs further clinical evaluation.

## Data Availability Statement

The original contributions presented in the study are included in the article/supplementary material, further inquiries can be directed to the corresponding author/s.

## Ethics Statement

Ethical review and approval was not required for the study on human participants in accordance with the local legislation and institutional requirements. The patients/participants provided their written informed consent to participate in this study. Written informed consent was obtained from the individual(s) for the publication of any potentially identifiable images or data included in this article.

## Author Contributions

SG and TF conceived the study and wrote the manuscript. TF, AC, CP, EB, and FV managed all the patients and initiated the proposal for subcutaneous therapy as suppressive treatment during multidisciplinary meetings. M-CG, SC, and JG performed drug therapeutic drug monitoring. SG performed PK/PD analysis of TDM data. FL provided microbiology results with MIC determination. All the authors participated in manuscript editing with significant intellectual inputs. All authors approved the final version of the manuscript.

## Conflict of Interest

The authors declare that the research was conducted in the absence of any commercial or financial relationships that could be construed as a potential conflict of interest.
